# Generation of Core–Sheath Polymer Nanofibers by Pressurised Gyration

**DOI:** 10.3390/polym12081709

**Published:** 2020-07-30

**Authors:** Suntharavathanan Mahalingam, Suguo Huo, Shervanthi Homer-Vanniasinkam, Mohan Edirisinghe

**Affiliations:** 1 Department of Mechanical Engineering, University College London, London WC1E 7JE, UK; suntharavathanan.mahalingam@ucl.ac.uk (S.M.); s.homer-v@ucl.ac.uk (S.H.-V.); 2 London Centre for Nanotechnology, University College London, London WC1H 0AH, UK; s.huo@ucl.ac.uk

**Keywords:** nanofiber, core, sheath, pressure, gyration

## Abstract

The ability to generate core–sheath bicomponent polymer nanofibers in a single-step with scale-up possibilities is demonstrated using pressurised gyration manufacturing. This is the first time that nanofiber containing more than one polymer having a core–sheath configuration has been generated in this way. Water-soluble polymers polyethylene oxide (PEO) and polyvinyl pyrrolidone (PVP) are used as the core and sheath layers, respectively. Core–sheath nanofibers with a diameter in the range of 331 to 998 nm were spun using 15 wt % PEO and 15 wt % PVP polymer solutions. The forming parameters, working pressure and rotating speed, had a significant influence on the size, size distribution and the surface morphology of the nanofibers generated. Overall, fibre size decreased with increasing working pressure and rotating speed. The fibre size was normally distributed in all cases, with 0.2 MPa working pressure in particular showing narrower distribution. The fibre size distributions for 0.1 and 0.3 MPa working pressure were broader and a mean fibre size of 331 nm was obtained in the latter case. The fibre size was evenly distributed and narrower for rotating speeds of 2000 and 4000 RPMs. The distribution was broader for rotating speed of 6000 RPM with a mean value obtained at 430 nm. Continuous, smooth and bead-free fibre morphologies were obtained in each case. The fibre cross-section analysis using a focused ion beam machine showed a solid core surrounded by a sheath layer. Our findings demonstrate that the pressurised gyration could be used to produce core–sheath polymer nanofibers reliably and cost-effectively with scale-up possibilities (~4 kg h^−1^).

## 1. Introduction

The applications of polymer nanofibers are boundless and have made a significant breakthrough in regenerative medicine, drug delivery, and electronic devices over two decades [1,2,3,4,5,6,7,8,9,10]. Polymer nanofibers were regarded as a beacon in the research arena connecting academia, engineers, clinicians, chemists, biologists, and many others, and continue to serve humankind fulfilling their specific needs. Core–sheath nanofibers are a variety of polymer nanofibers and comprise of a long continuous inner strand called a core, which is surrounded by an outer layer referred to as the sheath [11]. Designing core–sheath nanofibers using two or different polymer phases has numerous advantages compared to the single-phase nanofibers, enhancing the properties of the nanofiber (e.g., mechanical) or introducing a new property (e.g., functional, such as bioactivity) [12].

Modifying the nanofibers by incorporation of additional layer or layers can enhance the performances of functional scaffolds and architectures that are used in regenerative medicine to repair damaged tissues and develop artificial organs [13,14]. In addition, nanoparticles, drugs, proteins, peptides, growth factors, and other bioactive components could be embedded, coated, and encapsulated within the core–sheath structure and their use regulated more accurately in a time-programmed manner [15,16,17,18]. The flexibility of core–sheath nanofibers provide combining two or more modalities (e.g., photothermal drug delivery) for efficient, controlled and safe delivery of a bioactive molecule to the target site [19].

Electrospinning is a favourite and arguably the best technique to produce nanofibers. Many researchers have taken advantage of its versatility and easy to use aspects over many years despite its dependence on high-voltage [20]. It is widely claimed as a bed-rock methodology for regenerative medicine to fabricate functional tissue constructs combining scaffolds, signalling molecules, and living cells to repair damaged tissues in tendons, cartilage, bone, nerves, and the heart [21]. However, there are limitations in this technique, such as low cell infiltration where the constructed scaffolds predominantly behave like 2D sheets [22]. The difficulty of producing 3D scaffolds with exact dimensions and morphology has led to the post-treatment of scaffolds by methods such as leaching and laser machinery, but these were considered to be time-consuming and ineffective in achieving a 3D structure [22].

Pressurised gyration is an alternative electric field free production technique to process polymer nanofibers on a large scale in a single step by combining pressure and spinning of solute contained in a perforated aluminium vessel [23,24,25,26,27,28,29,30,31]. It is a straightforward and easy to use technique to create fibres and fibrous structures with a tailored fibre size and fibre size distribution [23]. Over a couple of years, pressurised gyration has gone through many developments, in particular the modulation of the forming parametric space is enormous. The process can control the flow rate of polymer solution to the vessel in the absence of pressure [25]. In addition, pressure-coupled infusion gyration renders the control of flow rate of material concurrently with pressure, and this process has been shown to form well-aligned nanofibers with excellent size control [26]. Moreover, melt pressurised gyration offers another dimension to this manufacturing method where extrusion of polymer pellets at high temperature is possible without the use of any hazardous solvents [27]. Recently, twin-reservoir pressurised gyration paved the way to process core–sheath fibres in the micrometre range with two different polymer phases subjected to simultaneous forming in an innovative spinneret [28].

In this work, we report the generation of core–sheath polymer nanofibers with a combination of two water-soluble polymers, polyethylene oxide (PEO) and polyvinyl pyrrolidone (PVP) by twin-reservoir pressurised gyration. The individual polymers (PEO and PVP) have been spun in single material pressirised gyration investigations and shown to generate nanofibers in our previous work [23,31]. Therefore, these two polymers are chosen for the present investigation based on their viscosities and formability for pressurised gyration to generate core–sheath nanofibers. The planar and cross-sectional microstructures of the generated core–sheath nanofibers were analysed. In addition, this study shows how the rotating speed and working pressure influence fibre formation and fibre size.

## 2. Experimental

### 2.1. Materials

Polyethylene oxide (PEO) (*M*_w_ = 200,000 g mol^−1^) and polyvinyl pyrrolidone (PVP) (*M*_w_ = 1,500,000 g mol^−1^) were obtained from Sigma Aldrich (Poole, UK). Deionised water was used as a solvent for PEO and PVP.

### 2.2. Solution Preparation and Characterisation

PEO powder was dissolved in deionised water in the weight ratio of 15:85 in an airtight bottle and magnetically stirred for 24 h at ambient temperature (~20 °C). Similarly, PVP powder was dissolved in deionised water in the weight ratio of 15:85 in an airtight bottle and magnetically stirred for 24 h at ambient temperature. The surface tension and viscosity of the polymer solutions were characterised using calibrated a Kruss tensiometer (Kruss GmbH, Hamburg, Germany) and a Brookfield viscosity meter (AMETEK Brookfield, Harlow, UK), respectively.

### 2.3. Experimental Set-Up

**Figure 1:**
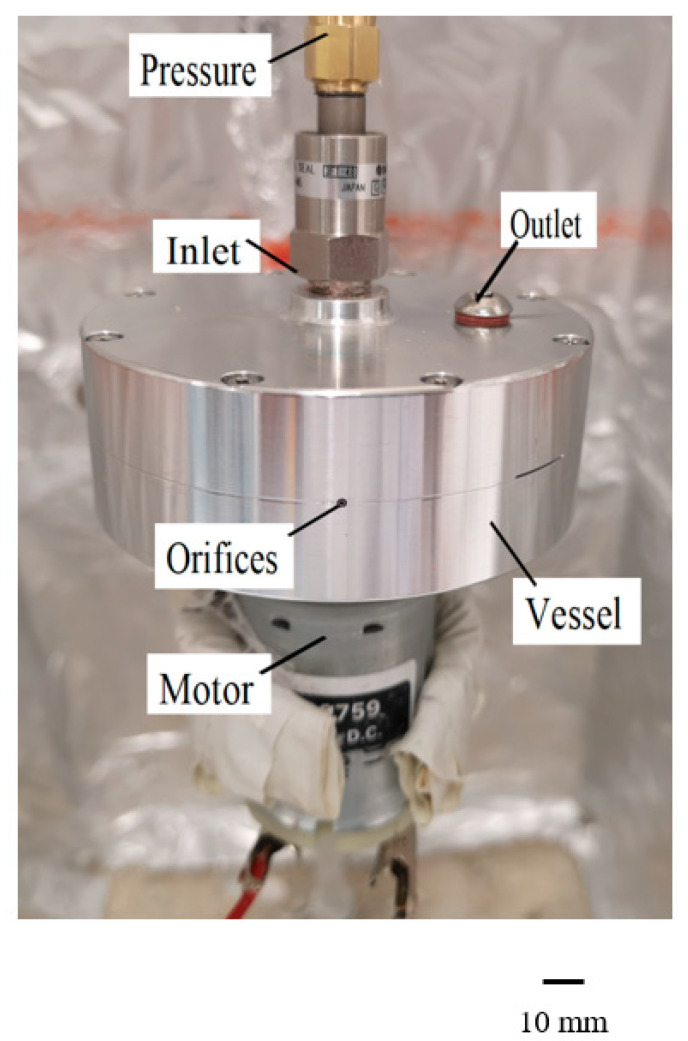
Pressurised gyration set-up.

The experimental set-up used to generate core–sheath nanofibers is shown in Figure 1 and full details are given in Reference 28. The design of the equipment used is found in our previous work [28]. It consists of a twin-reservoir aluminium cylindrical vessel of 100 mm and 80 mm, outer diameter and inner diameter, respectively. The inner vessel material loading capacity is 20 mL and the outer vessel capacity is 20 mL. In addition, there are two capillaries with a 1.6 mm outer diameter and a 0.8 mm inner diameter, respectively, attached to the vessel. Moreover, there are inlets and outlets at the top of the vessel to feed the polymer solutions into the vessels. One end of the vessel is connected to a DC motor and the other end of the vessel is connected to pressurised gas (N_2_). The rotational speed of the vessel can go up to 6000 RPM and the pressure can be elevated to 0.3 MPa. Before commencing the gyration experiments, the equipment was calibrated for its rotational speed. The generated fibres were collected on a stationary aluminium foil attached in the device mount 150mm away from the centre of spinning (Figure 1).

### 2.4. Core–Sheath Fibre Preparation

The core–sheath fibres were prepared by loading 5 mL of PEO polymer solution in the inner vessel and the 5 mL of PVP polymer solution in the outer vessel. Initially, they were spun at three different rotating speeds (2000, 4000 and 6000 RPM) and a fixed working pressure of 0.1 MPa. To evaluate the influence of working pressure on generation of core–sheath fibres, 15 wt % PVP and 15 wt % PEO polymer solutions were spun at different working pressure (0.1, 0.2, and 0.3 MPa) and a fixed rotating speed of 6000 RPM.

### 2.5. Characterisation of Core–Sheath Fibres

Scanning electron microscopy (FE-SEM, model JSM 6301 F, JEOL Inc, MA, USA) on the samples was carried out at 5 kV applied voltage. Before imaging, the samples were coated with gold using an Edwards Sputter S1 50B coater (Edwards Vacuum, London, UK) for 75 s. Fibre diameter was quantified for various forming conditions in the planar microstructure of the core–sheath nanofibers using Image J analysis. For this, more than 100 fibres were chosen at random points in the scanning electron microscopy images. Focused ion beam (FIB) milling and imaging were carried out on the samples to verify the core–sheath structure using a Zeiss NV40 FIB/SEM machine (Carl Zeiss, Oberkochen, Germany). The milling and polishing of the samples were achieved with a current of 150 pA and 80 pA, respectively. The images were obtained at 5 kV applied voltage.

## 3. Results and Discussion

### 3.1. Effect of the Polymer Solution’s Physical Properties on Formability

**Figure 2:**
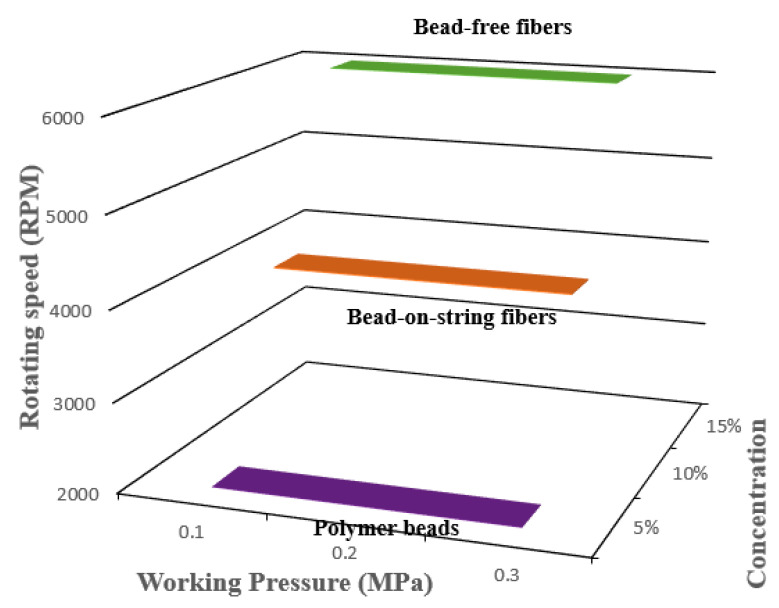
Product mapping as a function of forming parameters.

The polymer solution’s physical properties are important governing parameters of pressurised gyration in order to generate quality products. Thus, the viscosity and the surface tension are key processing parameters in pressurised gyration to generate defect-free fibres [29]. A higher polymer solution viscosity increases the physical chain interlocking of the polymers and is vital for stabilising the polymer jet, which, together with solvent evaporation, controls the process of fibre generation in pressurised gyration [30]. If solution viscosity is low, then the hydrodynamic volume of the polymer is low, which, together with an increase in surface tension, promotes bead formation. In this instance, the withholding surface tension overcomes the destabilising centrifugal force and the dynamic fluid flow to form polymer beads. If solution viscosity is too high, it will impose greater resistance against the destabilising centrifugal force and result in dynamic fluid blowing, which will promote the generation of thicker fibres, large beads or solidification [30]. Generally, increasing the polymer solution concentration increases the solution viscosity. Thus, it is crucial to find the right polymer solution concentration to form bead-free uniform polymer nanofibers. Figure 2 shows the product mapping of pressurised gyration with regard to processing parameters rotating speed, working pressure, and polymer concentration. In this work, the 5 wt % polymer concentration produced polymer beads, not fibres. Increasing the polymer concentration to 10 wt % generated fibres, but they showed bead-on-string morphology. A polymer concentration of 15 wt % resulted in bead-free nanofibers. Therefore, the polymer concentration (15 wt %) is selected as an optimum concentration to form bead-free, smooth cylindrical fibres [23,31]. The measured viscosities for 15 wt % PEO and 15 wt % PVP polymer solutions were 2200 and 3465 mPa s, respectively. The surface tension of the PEO solution was 52 mN/m, while it was 77 mN/m for the PVP solution. For same weight percentage (15%), both viscosity and surface tension values were higher for the PVP polymer solution than the PEO polymer solution. This is due to the difference in molecular weight: PVP has a greater molecular weight than the PEO used in the present experiments.

### 3.2. Effect of Working Pressure on Fibre Morphology

**Figure 3:**
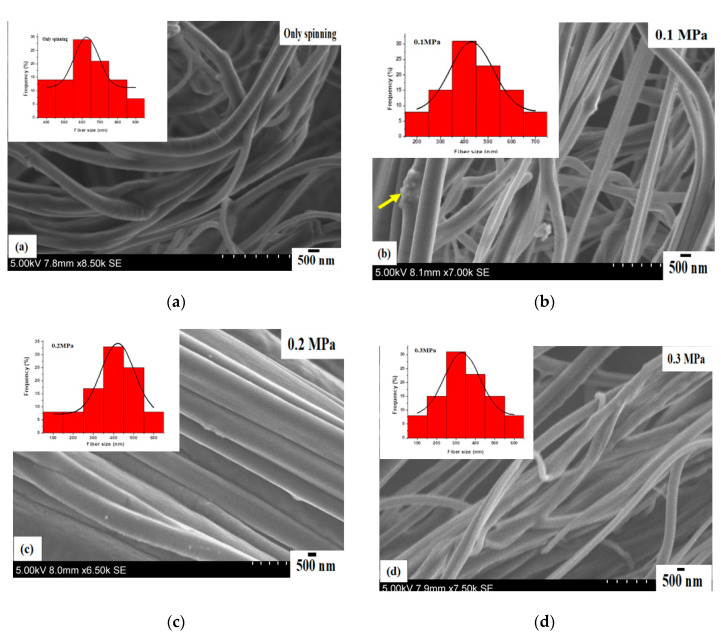
Scanning electron micrographs of core–sheath nanofibers obtained at a constant rotating speed 6000 RPM and different working pressures (MPa) (**a**) spinning only (**b**) 0.1 (**c**) 0.2 (**d**) 0.3. Insets show the corresponding fibre size distributions of core–sheath nanofibers for each case.

Figure 3 shows the scanning electron micrographs of core–sheath nanofibers obtained from a constant rotating speed of 6000 RPM and different working pressures. The nanofibers produced at no pressure (similar to centrifugal spinning) are randomly aligned, cylindrical in shape and are bent in some regions. The surface of the nanofibers appears rough and there are no apparent pores observed. The nanofibers are uneven along the axial direction (Figure 3a). Figure 3b shows core–sheath nanofibers obtained at 0.1 MPa working pressure. In this instance, the fibres are continuous, smooth and randomly aligned. In some regions, nanofibers are fused. The nanofibers are bread-free, however, there were polymer clumps (see arrow) in some regions. The nanofibers produced at 0.2 MPa working pressure are shown in Figure 3c, and are continuous, smooth and bead-free. The fibres are layered and closely packed together. There are no apparent pores seen on the surface and nanofibers are even along the axial direction. Figure 3d displays the core–sheath nanofibers formed at 0.3 MPa working pressure. The fibres are continuous, smooth and bead-free. They are randomly aligned and there are no visible pores on the surface.

### 3.3. Effect of Working Pressure on Fibre Size and Size Distribution

Figure 3 insets exhibit the fibre size distributions of core–sheath nanofibers obtained at a constant rotating speed of 6000 RPM and different working pressures. The fibre size is normally distributed and unimodal with one maximum and broad width. The mean fibre diameter is 625 nm with a standard deviation of 68 nm for fibres produced with no pressure (Figure 3a). For 0.1 MPa working pressure, the fibre size distribution exhibits unimodal normal distribution. In this instance, the mean fibre diameter decreased to 430 nm with a standard deviation of 91 nm (Figure 3b). The fibre size distribution is negatively skewed and displayed unimodal characteristics for 0.2 MPa working pressure (Figure 3c). In this case, the mean fibre diameter is 390 nm with a standard deviation of 82 nm. For 0.3 MPa working pressure, the fibre size distribution is normally distributed and the mean fibre diameter obtained is 331 nm, with a standard deviation of 90 nm (Figure 3d). The fibre size distribution in Figure 3a–d gives polydispersities of 11%, 21%, 21%, 27% for a rotating speed of 6000 RPM and a working pressure range of 0.1 to 0.3 MPa. Overall, there is a decreasing trend for mean fibre diameter when increasing the working pressure at a constant rotating speed. This is due to the introduced gas stream which enhances the combined shearing force consisting of centrifugal force and blowing against the surface tension force causing elongational flow of polymer jets, thereby reducing the diameters of the ejected polymer jets at the orifices of the vessel, which helps to promote thinner fibre formation [23]. Solvent evaporation can be enhanced by blowing, which affects the relative speed of the gas flow on the liquid–air interface in the polymer drops at the orifices as well as solvent diffusion across the polymer drops to the surface, thus contributing to the thinning of the fibres [26].

### 3.4. Yield of the Core–Sheath Nanofibers

The yield of the core–sheath nanofibers obtained here has been compared with other manufacturing techniques, as shown in Table 1. Core–sheath pressurised gyration has a higher yield than centrifugal spinning and electrospinning techniques (Table 1). However, it has a lower yield than solution blowing and single material pressurised gyration.

Here core–sheath nanofibers were obtained by choosing two water-soluble polymers, PEO and PVP, as the core and the sheath fluids, respectively. The water-soluble polymer solutions had higher viscosities compared to non-aqueous polymers used in our earlier work [28]. The viscoelasticity of polymer solution is a well-known property of any polymer system expressing time-dependent elastic and viscous responses to an external force. The time constant is a parameter during which the external force acting on the polymer system governs the polymer jet stretching in the pressurised gyration process. The time constant is a function of viscosity. If the time constant is short, the polymer jet exhibits a more elastic response where polymer chains rearrange themselves very quickly. If the time constant is long, then the polymer jets exhibit a more viscous response where the polymer chains undergo deformation/stretching with respect to an external force. The time constant of the polymer solutions varies between one-hundredth of a second to one-tenth of a second. Solvent evaporation is another parameter which strongly influences fibre formation in pressurised gyration [34]. Higher boiling point solvents like water evaporate slowly from the ejected fibre jets during spinning, causing viscoelastic properties to change and, hence, the stretching of the polymer fibre jets. It is also noteworthy that dynamic fluid blowing will accelerate the solvent evaporation during spinning. The polymer jets leaving the orifices experience greater elongational stress due to dynamic fluid flow, which significantly affects molecular stretching. The change in velocity along the fluid flow streamlines and the difference in velocity at the ends of the molecular chain reduces the cross-sectional area of the fibres drawn. Thus, by selecting two water-soluble polymers, we have provided a greater visco-response in the polymer jets combined with slower evaporation and stretching from the ejected polymer jets to generate the core–sheath nanofibers.

In addition, PEO and PVP could be swapped as the sheath and core, respectively, to form the core–sheath nanofibers. The structures of PEO and PVP are different: PEO is an ether and PVP is a ketone. The molecular weight of these polymers are also different, but both are water-soluble polymers. The obtained viscosities for 15 wt % of PVP are higher than the 15 wt % PEO, even though they have the same number of solutes in a given amount of solvent. However, PVP will evaporate more slowly than PEO since it has a higher viscosity. The rate of evaporation also depends on other factors, such as temperature, humidity and blowing of gas. The temperature and humidity in this work are fixed (20 °C, 42%) so the only factor that will affect the rate of evaporation is the blowing of gas. It should also be noted that the temperature of the vessel can go up during gyration, but the rise in temperature in water is not overly significant (1–2 °C) to causes a dramatic change in viscosity.

If PEO is the sheath fluid, it will evaporate more quickly than the PVP core fluid and will cause the encapsulation of the inner core. Moreover, the selection of water-soluble polymers must be based on molecular weight and viscosity. The molecular weight of the polymer affects the concentration of the polymer solution, and by extension the viscosity of the solution. The greater the molecular weight, the higher the viscosity for a given polymer concentration. The polymer chain entanglement increases with an increasing molecular weight and viscosity. The other factor we must consider is the formability of the polymer. The polymer chains must deform/stretch during spinning to make fibres. For example, gelatine is not soluble in cold water, but it is soluble in hot water. If it is cooled, it will become a gel. Gels have higher viscosity, but their excessive viscosity make formability poor. Therefore, the selection of polymers must take into account the viscosity and formability of polymers.

### 3.5. Applications of Water-Soluble Polymers

Water-soluble polymers are used in drug delivery and tissue engineering due to their cost-effectiveness, biocompatibility and non-toxicity. However, the hydrophilic and hygroscopic properties make them weaker in handling pressure. Other water-soluble polymers like hydroxyethyl cellulose (HEC) are used in the biomedical arena, paint industry, soil amendments (they improve permeability and water retention) in agriculture, coal dewatering, cosmetics, wastewater treatment, absorbent pads and gel electrolyte membranes (they have good thermal stability, good cycling stability and high capacity retention). Moreover, water-soluble polymers could be made as a compound and grafted with other polymers to modify their physical/chemical/biological properties, e.g., combining hydrophilic and hydrophobic polymers for controlled drug release, for example grafting HEC with polylactic acid to functionalise the main polymer chain to alter the properties [35,36,37].

### 3.6. Effect of Rotating Speed on Fibre Morphology, Fibre Size and Size Distribution

**Figure 4:**
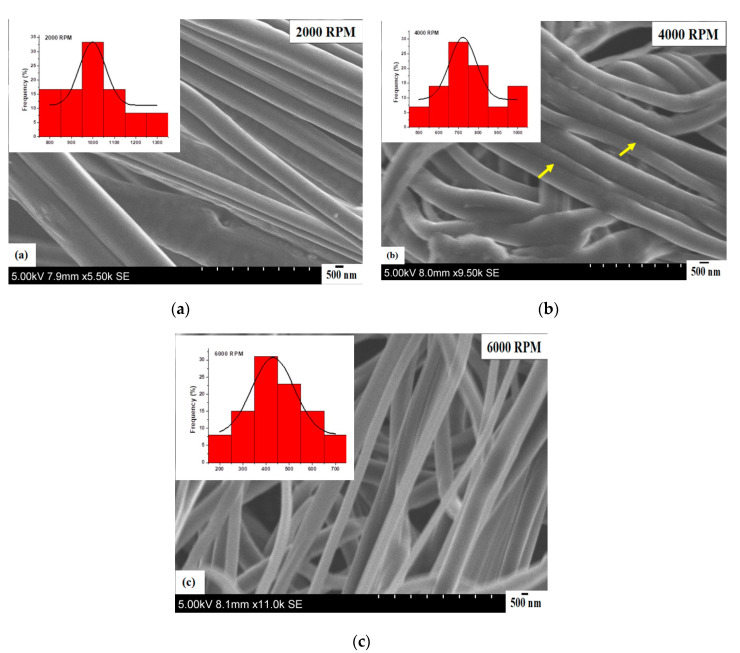
Scanning electron micrographs of core–sheath nanofibers obtained at a constant working pressure of 0.1 MPa and different rotating speeds (**a**) 2000 RPM (**b**) 4000 RPM (**c**) 6000 RPM. Insets show the corresponding fibre size distributions of core–sheath nanofibers for each case.

Figure 4 shows the scanning electron micrographs of core–sheath nanofibers obtained at a constant working pressure of 0.1 MPa and at different rotating speeds. The nanofibers were continuous, smooth and had no bead structures on their surface at a rotating speed of 2000 RPM. In addition, they were well-aligned and closely packed together (Figure 4a). While the core–sheath nanofibers are fused at some points (see arrows) at a rotating speed of 4000 RPM, they are smooth and bead-free (Figure 4b). The nanofibers were continuous, smooth and bead-free at a rotating speed of 6000 RPM (Figure 4c). Figure 4 insets show unimodal fibre size distributions for all of these cases. The fibre size is normally distributed for nanofibers spun at 2000 RPM. The mean fibre diameter is located at 998 nm with a standard deviation of 61 nm (Figure 4a). For 4000 RPM, the fibre size is a normal distribution with a maximum (mean) found at 720 nm; here, the standard deviation is 67 nm (Figure 4b). The fibre size is normally distributed with an even spread of nanofibers spun at 6000 RPM. The mean fibre diameter is 430 nm with a standard deviation of 91 nm (Figure 4c). The nanofibers obtained at rotating speeds of 2000 and 4000 RPM are very similar and display narrow distribution (polydispersities of 6% and 9%, respectively), whereas the nanofibers spun at 6000 RPM show a broader fibre size distribution (polydispersity of 21%).

### 3.7. Focused Ion Beam Imaging and Analysis

**Figure 5:**
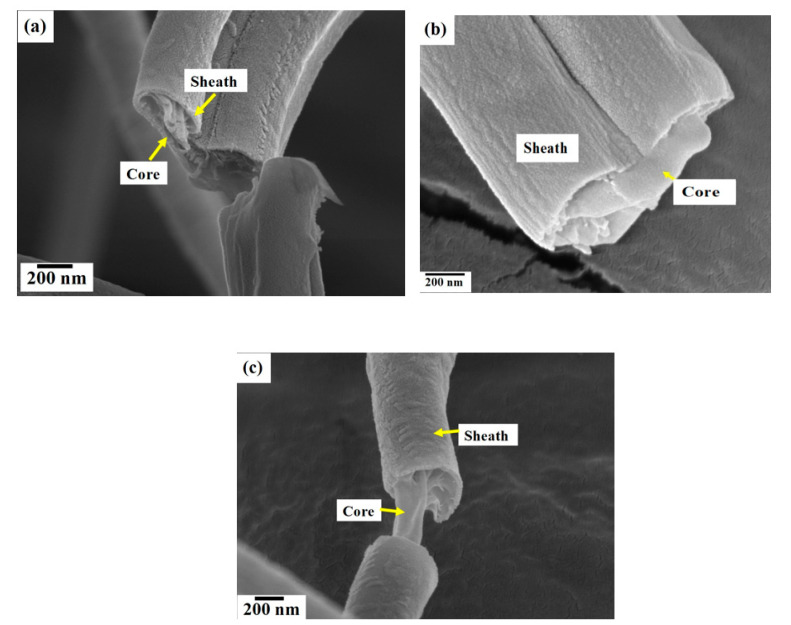
Core–sheath nanofiber cross-sections uncovered using focused ion beam milling. Rotating speed of 6000 RPM and different working pressures (MPa) (**a**) 0.1 (**b**) 0.2 (**c**) 0.3.

**Figure 6:**
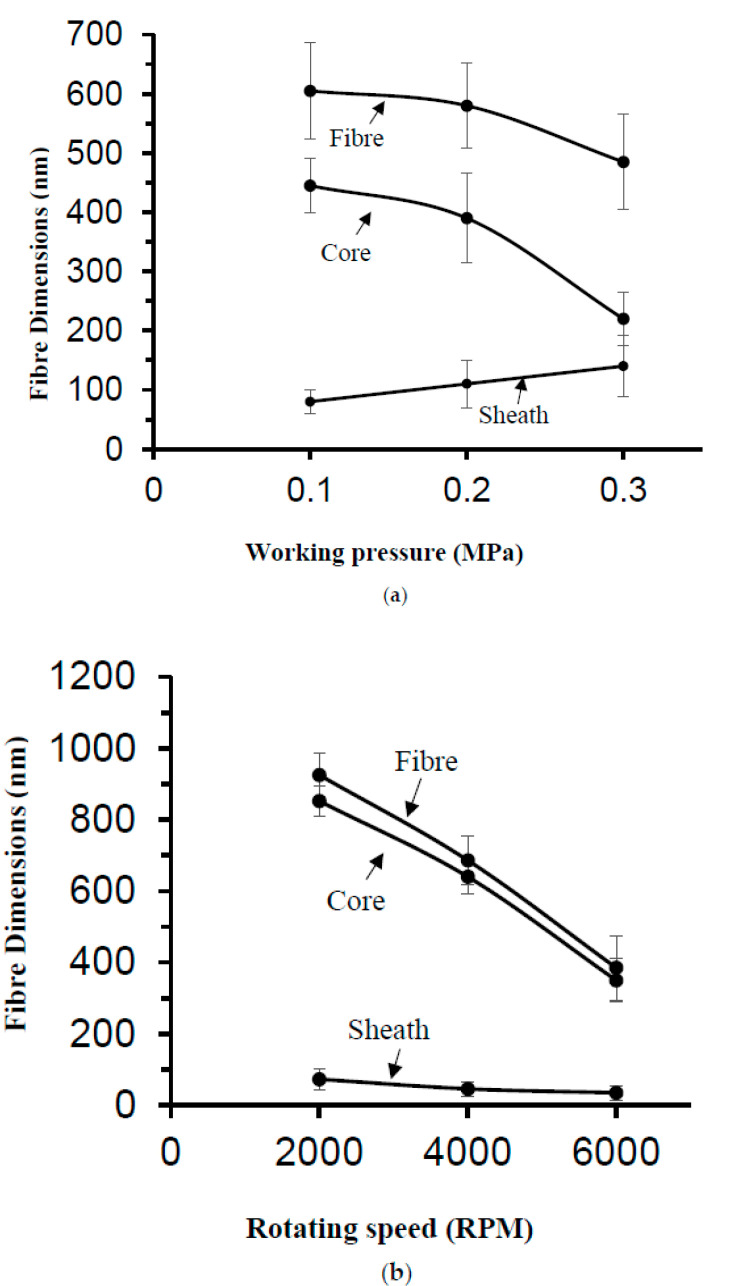
Fibre D2, core D1 and sheath (D2-D1)/2 dimensions as a function of (**a**) working pressure at a constant rotating speed of 6000 RPM (**b**); a rotating speed at a constant working pressure of 0.1 MPa.

To further examine the fibre morphology and to verify core–sheath bicomponent formation, the nanofibers produced at a constant rotating speed 6000 RPM and different working pressures were analysed using focused ion beam milling and imaging. Figure 5 shows the core–sheath nanofiber cross-sections obtained at different forming conditions. Figure 5a displays the fibre cross-section obtained at a 0.1 MPa working pressure and a rotating speed of 6000 RPM, and it is clearly seen that the core PEO layer is sandwiched inside the PVP sheath layer. The fractured PEO core layer is protruding outside the rounded sheath PVP structure. The cross-section appears rougher compared to the surface of the nanofibers, but this is due to the ion milling. Figure 5b displays the fibre cross-section obtained at a 0.2 MPa working pressure. The cross-section appears flat with a solid core section surrounded by a curved sheath layer. The surface of the core is smoother than the surface of the sheath layer and there are no pores observed on the surface of the nanofibers. Figure 5c shows the fibre cross-section obtained at a 0.3 MPa working pressure and a rotating speed of 6000 RPM. In this instance, the core layer is pulled and stretched along the axial direction. There are no pores observed on both the cross-section and on the surface of nanofibers. Figure 6 shows the fibre dimensions as a function of working pressure and rotating speed. The approximate sheath width is obtained as follows: fibre diameter = D2, core diameter = D1, sheath width = (D2 − D1)/2. Increasing the working pressure reduces the core fibre diameter, while the sheath width increases with pressure (Figure 6a). This is due to an increase in working pressure which drives more polymer solution to the outer capillary. Increasing the rotating speed reduces the core fibre diameter and the sheath width (Figure 6b). In all cases, the core fibre diameter is higher than the sheath width. At a working pressure of 0.3 MPa, the core fibre diameter and the sheath width convergences indicate thinning of the sheath and non-existence of a core. The orifice diameter of the core fluid is half the value of the orifice diameter of the sheath fluid; however, the effective orifice diameter of the sheath is the same. The core and sheath fluids were separated by the thin wall of the cylindrical vessel, and there is a limited chance they will interact within the vessel during spinning. The sheath fluid experiences greater centrifugal force than the core fluid due to the greater radius of the outer vessel compared with the inner vessel and therefore acts as a driver of the core fluid when the fluids reach the orifices. Moreover, the sheath fluid is in contact with the external environment (e.g., N_2_) and will shield the core fluid. Thus, the sheath fluid evaporates more quickly than the core fluid, but both have the same boiling point and encapsulate the core fluid to form nanofibers. The interaction between the core and sheath fluid jets during spinning is negligible, and as it is a rapid process, it does not provide sufficient time for two fluids to diffuse into each other. Moreover, miscible solvents (water) will reduce the interfacial surface tension during spinning and produce thinner fibres.

## 4. Conclusions

In this study, core–sheath nanofibers containing a polyethylene oxide core and polyvinyl pyrrolidone sheath layers were fabricated using novel pressurised gyration manufacturing technology. The effect of the processing conditions such as rotating speed and working pressure on fibre size and fibre size distribution were analysed. Overall, the fibre size decreased with increasing working pressure at a constant rotating speed. The fibre size was normally distributed for no pressure (spinning only), 0.1 and 0.3 MPa. The fibre size distribution obtained at a working pressure of 0.2 MPa showed a skewed distribution. The rotating speed had a significant influence on the fibre diameter. The fibre size decreased with increasing rotating speed. The fibre size was evenly distributed for all the rotating speeds used in this study. Overall, the surface morphology of the nanofibers revealed continuous, smooth and bead-free structures. Focused ion beam analysis confirmed core–sheath bicomponent structure formation.

## Figures and Tables

**Table 1:** Comparison of core–sheath polymer yield.

Technique	Yield (kg h^−1^)
Centrifugal spinning [32].	0.1
Solution blowing [33].	7–8
Electrospinning [32].	0.2
Single material pressurised gyration (PEO) [23].	6
Core–sheath pressurised gyration (PEO–PVP)	4.2
